# Healthcare Provider Recommendations and Observed Changes in HPV Vaccination Acceptance during the COVID-19 Pandemic

**DOI:** 10.3390/vaccines10091515

**Published:** 2022-09-12

**Authors:** Ikponmwosa Osaghae, Onyema Greg Chido-Amajuoyi, Sanjay Shete

**Affiliations:** 1 Department of Biostatistics, The University of Texas MD Anderson Cancer Center, Houston, TX 77030, USA; 2 Division of Cancer Prevention and Population Sciences, The University of Texas MD Anderson Cancer Center, Houston, TX 77030, USA; 3 Department of Epidemiology, Human Genetics and Environmental Sciences, The University of Texas Health Science Center at Houston (UTHealth), School of Public Health, Houston, TX 77030, USA; 4 Department of Epidemiology, The University of Texas MD Anderson Cancer Center, Houston, TX 77030, USA

**Keywords:** HPV vaccination, COVID-19, healthcare provider, provider recommendation, vaccination acceptance

## Abstract

Healthcare provider (HCP) recommendation of the human papillomavirus (HPV) vaccination is crucial for HPV vaccination acceptance and uptake. It is unclear to what extent the disruptive effect of the COVID-19 pandemic impacted the recommendation and acceptance of HPV vaccination. HCPs practicing in Texas were invited to complete an online survey between January and April 2021. This population-based survey examined the association between HPV vaccination recommendation by HCPs and their observed changes in HPV vaccination acceptance during the COVID-19 pandemic. Of the total 715 HCPs included in this study, 13.9% reported a decrease, 8.7% reported an increase, and 77.5% reported no change in HPV vaccination acceptance during the COVID-19 pandemic. Compared to the HCPs who never/sometimes recommend HPV vaccination, those who often/always recommend HPV vaccination were less likely to observe a decrease (12.3% vs. 22.1%) and more likely to observe an increase in HPV vaccination (9.1% vs. 6.2%), during the COVID-19 pandemic. Furthermore, those who provided recommendations often/always had 46% (odds ratio: 0.54; 95%CI: 0.30–0.96) lower odds of reporting a decrease in HPV vaccination acceptance during the COVID-19 pandemic. This study adds to prior evidence of the positive influence of provider recommendations on HPV vaccination acceptance despite the disruptive effect of the COVID-19 pandemic on cancer prevention services.

## 1. Introduction

Human papillomavirus (HPV) is a group of double-stranded DNA viruses attributable to several diseases and cancers [1,2]. HPV infections are common, with most persons at risk of having an HPV infection in their lifetime [1,3]. The risk of acquiring HPV infection also increases with the number of lifetime sexual partners [3]. In the U.S., there are over 42 million individuals with HPV infections, with about 13 million new infections yearly [4]. Whereas low-risk HPV is linked with anogenital warts, high-risk HPV types have been associated with six cancers, including cancers of the cervix, vulva, vagina, anus, penis, and throat [1,2,5]. Globally, about 650,000 cases of cancers are attributable to HPV annually [2,6]. In the U.S., about 45,000 HPV-associated cancers are reported annually, with HPV accounting for 80% of these cancers [7].

To control HPV infections and reduce HPV-associated diseases and cancers, HPV vaccination has proven to be safe and effective [8,9,10,11]. The first HPV vaccine was licensed in 2006 for use in females and in 2009 for use in males as a public health strategy to mitigate the rising trends in HPV infections and associated diseases [12]. The HPV vaccine is routinely recommended by the U.S. Centers for Diseases Control Advisory Committee for Immunization Practices (ACIP) for males and females aged 11–12 years [13]. The ACIP further recommends that HPV vaccines could be administered as early as nine years and up to age 26 years for everyone not adequately vaccinated [13]. However, in the US and Texas, HPV vaccination rates remain suboptimal and are behind global and national targets [14,15,16]. Since licensure, although the HPV vaccination rate in the U.S. increased, reaching 59% in 2020, it is still lower than the rates for other childhood vaccinations [14,17]. Similarly, in Texas, the second largest state in the US, the HPV vaccination rate (54%) ranks 40th in the nation [14].

The COVID-19 pandemic was accompanied by a disruption in access to care, clinic appointments, and the delivery of routine vaccines [18,19,20]. For example, in 2020, following the COVID-19 pandemic, women were less likely to receive preventive screening services for cervical cancer and sexually transmitted infections than the year before [21]. In addition, childhood vaccination rates decreased at the onset of the COVID-19 pandemic compared to the preceding two years [18]. Specifically, following the COVID-19 pandemic, there was a decline in the doses of HPV vaccines administered in the U.S. during the first quarter of 2020 compared to the same period in the preceding two years [22].

Moreover, HPV vaccine hesitancy by parents and patients had increased even before the onset of the pandemic, and this is a known barrier to HPV vaccination uptake [23,24,25,26]. Studies have found that healthcare provider (HCP) recommendation of vaccines is an effective strategy to increase vaccination acceptance and is associated with increased HPV vaccination initiation and completion rates [27,28]. However, the disruptions in clinical practice seen during the pandemic may have eroded past gains achieved from HCP recommendations of HPV vaccination before COVID-19 [17]. While it is known that HCP recommendation of HPV vaccination is crucial for uptake, there is limited data on the effect of provider HPV vaccination recommendation on changes in HPV vaccination acceptance rates within the context of the COVID-19 pandemic. Therefore, the objective of this study was to examine the association between HPV vaccination recommendations by HCPs and their observed changes in HPV vaccination acceptance during the COVID-19 pandemic using data from a population-based survey of frontline HCPs.

## 2. Methods

### Study Setting, Population, and Data Collection

We conducted a cross-sectional study of HCPs practicing in Texas between January and April 2021, about a year into the COVID-19 pandemic. HCPs were defined as physicians (including pediatricians, family physicians, gynecologists, and internal medicine physicians), physician assistants, and nurse practitioners in Texas. Email addresses of HCPs were retrieved from the LexisNexis reference database under an annual license [29]. All Texas HCPs with email addresses available in the LexisNexis provider database were invited to complete an online survey developed by The University of Texas MD Anderson Cancer Center. Of the 1283 HCPs who completed the survey, 715 completed the question on HPV vaccination acceptance during the COVID-19 pandemic. There was no significant difference between respondents and non-respondents to the main survey question on observed changes in HPV vaccination acceptance during the COVID-19 pandemic with respect to the HCPs’ sex, race/ethnicity, provider type, and facility type. All participants provided informed consent to participate in the study. The study was approved by The University of Texas MD Anderson Cancer Center Institutional Review Board (IRB Number: 2019–1257). This study followed the Strengthening the Reporting of Observational Studies in Epidemiology (STROBE) guidelines [30].

## 3. Measures

### 3.1. Dependent Variable

#### Observed Changes in HPV Vaccination Acceptance

The dependent variable was the HCPs’ observed changes in HPV vaccination acceptance during the COVID-19 pandemic. In the survey, the HCPs responded to whether they observed changes in HPV vaccination acceptance during the COVID-19 pandemic. Possible responses provided were “Increased”, “Decreased”, “No-change”, or “Not Sure” (see Supplementary Material). Those who reported “Not Sure” (19%) were excluded from the study. The referent category was “No-Change”.

### 3.2. Independent Variable

#### HCP’s Recommendation of HPV Vaccination

The independent variable was an HCP’s recommendation of HPV vaccination. This was assessed based on the survey question, “For the unvaccinated, or incompletely vaccinated for HPV, do you recommend HPV vaccination?” Possible responses provided were “Never”, “Sometimes”, and “Often/Aways”. This was operationalized as a binary variable and recategorized as “Often/Always” versus “Never/Sometimes” (see Supplementary Material). The referent category was “Never/Sometimes”.

### 3.3. Covariates

The following covariates were included based on current literature and relevance to the study question: the HCPs’ age, sex, race/ethnicity, region of practice, number of years in practice, number of patients seen, provider type, and facility type. We created the variable region of practice from self-reported zip codes where the HCPs primarily work. We then linked the Federal Information Processing Standard (FIPS) Codes with the 2013 Rural-Urban Continuum Code (RUCC) developed by the U.S. Department of Agriculture [31]. RUCC codes ranging from one to nine were dichotomized, with one to three defined as urban and four to nine defined as rural.

## 4. Data Analysis

We described the distribution of the HCPs by strata of observed changes in HPV vaccination during the COVID-19 pandemic using frequency and proportion. We predetermined a priori covariates to include in our analyses based on the current literature and relevance to our study objective. Thus, we did not conduct any variable selection. Multivariable multinomial logistic regression analysis was used to assess the association between the recommendation of HPV vaccination and observed changes in HPV vaccination acceptance during the COVID-19 pandemic. Multinomial logistic regression models were adjusted for the HCPs’ age, sex, race/ethnicity, region of practice, number of years in practice, number of patients seen, provider type, and facility type. Statistical significance was set as a two-sided *p* < 0.05. All analyses were conducted in Stata/IC V.15.1.

## 5. Results

Of the 715 HCPs included in this study, 554 (77.5%) reported no change, 99 (13.9%) reported a decrease, and 62 (8.7%) reported an increase in HPV vaccination acceptance during the COVID-19 pandemic. A decrease in HPV vaccination acceptance during the COVID-19 pandemic was observed by 12.3% of the HCPs who often/always recommended HPV vaccination. In contrast, such a decrease was observed by 22.1% of the HCPs who never/sometimes recommended HPV vaccination. On the other hand, an increase in HPV vaccination acceptance during the COVID-19 pandemic was observed by 9.1% of HCPs who often/always recommend HPV vaccination. In comparison, such an increase was reported by 6.2% of the HCPs who never/sometimes recommend HPV vaccination. Furthermore, no change in HPV vaccination acceptance during the COVID-19 pandemic was observed in 78.6% of the HCPs who often/always recommend HPV vaccination, whereas no change in HPV vaccination acceptance was observed in 71.7% of the HCPs who never/sometimes recommend HPV vaccination (Table 1).

Following multivariable multinomial regression analysis (Table 2), we found that compared to the HCPs who never/sometimes recommend HPV vaccination, those who provided recommendations often/always had lower odds of reporting a decrease in HPV vaccination acceptance during the COVID-19 pandemic relative to reporting no change (adjusted odds ratio (AOR): 0.54; 95% CI: 0.30–0.96). In addition, compared to the HCPs who never/sometimes recommend HPV vaccination, those who provided recommendations often/always had higher odds of reporting an increase in HPV vaccination acceptance during the COVID-19 pandemic relative to reporting no change while adjusting for other covariates (AOR: 1.24; 95% CI: 0.52–2.96). However, the latter association did not reach statistical significance (Table 2).

## 6. Discussion

In this study, HCP recommendation of HPV vaccination was associated with lower odds of HCPs observing declines in acceptance of HPV vaccination during the COVD-19 pandemic. Findings from this study add to significant evidence of the positive influence of HCP recommendations on HPV vaccination acceptance and uptake [27,28]. Before the pandemic, several studies have shown that HCP recommendation of HPV vaccination is positively associated with increased vaccination rates [27,28,32]. For example, a systematic review and meta-analysis of studies published between 2006 and 2009 found that HCP recommendation of HPV vaccination was associated with increased HPV vaccination initiation, completion, and follow-through [27]. Our finding, however, further points to the importance of provider recommendations on acceptance of HPV vaccination despite the COVID-19 pandemic.

Importantly, this study is unique in that it documents this association from the vantage point of frontline HCPs during a pandemic. HCPs play an essential role in patients’ safe health decisions regarding vaccination since patients tend to value the opinion of their HCPs [33]. Gilkey et al. found an 11-fold increase in the decision to receive HPV vaccination if it was offered by an HCP [34]. However, the COVID-19 pandemic disrupted access to routine healthcare services such as office visits to primary care providers and clinic appointments to administer vaccines. From an HPV vaccination standpoint, there was concern that this disruption in access to HCPs may lead to declines in physician recommendations and negatively impact HPV vaccination acceptance and uptake rates during the COVID-19 pandemic. However, the findings of this study suggest that the strong relationship between HCP recommendations and HPV vaccination held true despite the disruptive qualities of the pandemic. 

Amidst HPV vaccine hesitancy before the pandemic and the vaccine misinformation that heralded the COVID-19 pandemic, our study shows that provider recommendation is crucial for accepting HPV vaccination [24,35,36,37]. HCPs should continue recommending HPV vaccination at every clinical opportunity to increase HPV vaccination rates. Additionally, the perceived severity and increased threat following the COVID-19 pandemic may have increased patients’ perceived desirability and benefits from COVID-19 vaccines and other routine vaccines, including the HPV vaccine [38,39,40]. This perceived threat and desirability to adopt protective health behavior that accompanied the pandemic may have motivated patients to accept HPV vaccination when recommended by their providers.

This study has some limitations. This is a cross-sectional study; as such, we are unable to infer causality. Furthermore, the study is prone to potential information bias, given that HPV vaccination acceptance was based on the HCP’s recall of observed changes in HPV vaccination acceptance during the pandemic. However, the study used statewide representative data collected at the height of the COVID-19 pandemic from frontline HCPs, increasing the generalizability of the results. Thus, this study provides valuable insights into changes in HPV vaccination acceptance during the COVID-19 pandemic to support the implementation of interventions that aim at increasing the recommendation and uptake of HPV vaccination during and after the pandemic.

In conclusion, the HCPs who recommended HPV vaccination were less likely to observe a decrease in HPV vaccination acceptance during the COVID-19 pandemic. Hence, HCPs should continue recommending HPV vaccination to their patients at every clinical encounter during and after the pandemic to increase HPV vaccination acceptance and uptake. 

## Figures and Tables

**Table 1:** Distribution of recommendation and provider-related characteristics by observed changes in HPV vaccination acceptance during the COVID-19 pandemic.

Characteristics	Change in HPV Vaccine Acceptance (n =715)		
	Decreased (n = 99)	Increased (n = 62)	No-Change (n = 554)
**Recommendation, n (%)**			
Never/sometimes	25 (22.1)	7 (6.2)	81 (71.7)
Often/always	74 (12.3)	55 (9.1)	473 (78.6)
**Provider age, years, n (%)**			
<35	10 (12.8)	7 (9.0)	61 (78.2)
35–54	59 (13.4)	42 (9.5)	341 (77.2)
≥55	30 (15.8)	12 (6.3)	148 (77.9)
**Sex, n (%)**			
Female	69 (12.8)	46 (8.5)	425 (78.7)
Male	29 (17.7)	15 (9.2)	120 (73.2)
**Region of practice, n (%)**			
Rural	6 (20.0)	2 (6.7)	22 (73.3)
Urban	93 (13.6)	60 (8.8)	531 (77.6)
**Race/ethnicity, n (%)**			
Non-Hispanic White	48 (13.3)	29 (8.1)	283 (78.6)
Non-Hispanic Black	10 (15.9)	7 (11.1)	46 (73.0)
Hispanic	19 (17.9)	9 (8.5)	78 (73.6)
Non-Hispanic other	18 (10.7)	16 (9.5)	135 (79.9)
**Provider type, n (%)**			
**Non-physician**	55 (16.8)	26 (8.0)	246 (75.2)
**Physician**	44 (11.3)	36 (9.3)	308 (79.4)
**Type of practice, n (%)**			
University/teaching hospital	23 (15.5)	10 (6.8)	115 (77.7)
Solo practice	14 (14.7)	7 (7.4)	74 (77.9)
Group practice	34 (11.9)	25 (8.7)	228 (79.4)
FQHC/public facility	15 (15.6)	13 (13.5)	68 (70.8)
Other	13 (14.6)	7 (7.9)	69 (77.5)
**Years in practice, n (%)**			
≤10 years	39 (15.5)	24 (9.6)	188 (74.9)
11–20 years	27 (10.9)	20 (8.1)	200 (81.0)
>20 years	33 (15.6)	16 (7.6)	163 (76.9)
**No. of patients seen (per week), n (%)**			
≤50	34 (14.1)	20 (8.3)	188 (77.7)
51–100	45 (12.8)	28 (7.9)	280 (79.3)
>100	18 (16.7)	13 (12.0)	77 (71.3)

**Table 2:** Multinomial logistic regression analysis of the association between provider recommendation and observed changes in HPV vaccination acceptance during the COVID-19 pandemic.

Characteristics	Decreased Versus No-Change		Increased Versus No-Change	
	aOR	95% CI	aOR	95% CI
**Recommendation**				
Never/sometimes	Ref	Ref	Ref	Ref
Often/always	0.54	0.30–0.96	1.24	0.52–2.96
**Provider age, years**				
<35	Ref	Ref	Ref	Ref
35–54	1.27	0.57–2.84	1.04	0.40–2.68
≥55	1.58	0.56–4.49	0.63	0.17–2.31
**Sex**				
Female	Ref	Ref	Ref	Ref
Male	1.48	0.86–2.53	1.17	0.61–2.26
**Region of practice**				
Rural	Ref	Ref	Ref	Ref
Urban	0.59	0.22–1.58	1.27	0.28–5.71
**Race/ethnicity**				
Non-Hispanic White	Ref	Ref	Ref	Ref
Non-Hispanic Black	1.36	0.62–2.98	1.20	0.46–3.15
Hispanic	1.35	0.73–2.52	0.99	0.44–2.22
Non-Hispanic other	0.90	0.49–1.65	0.95	0.48–1.88
**Provider type**				
Non-physician	Ref	Ref	Ref	Ref
Physician	0.65	0.40–1.06	1.11	0.62–2.00
**Type of practice**				
University/teaching hospital	Ref	Ref	Ref	Ref
Solo practice	0.83	0.38–1.80	1.23	0.42–3.57
Group practice	0.67	0.35–1.26	1.31	0.57–3.02
FQHC/public facility	1.01	0.47–2.16	2.24	0.86–5.86
Other	0.87	0.39–1.95	1.38	0.48–3.94
**Years in practice**				
≤10 years	Ref	Ref	Ref	Ref
11–20 years	0.60	0.33–1.10	0.82	0.41–1.68
>20 years	0.78	0.35–1.71	1.05	0.41–2.70
**No. of patients seen (per week)**				
≤50	Ref	Ref	Ref	Ref
51–100	1.05	0.63–1.76	0.87	0.46–1.65
>100	1.27	0.61–2.66	1.65	0.72–3.75

aOR = adjusted odds ratio; multivariable multinomial logistic regression analysis was adjusted for the HCP’s age, sex, race/ethnicity, region of practice, provider type, type of practice, years in practice, and number of patients seen. CI = confidence interval; Ref = reference; FQHC = Federally Qualified Health Center.

## Data Availability

The data related to this manuscript can be obtained, on a reasonable request, from the corresponding author.

## Supplementary Materials

The following supporting information can be downloaded at: https://www.mdpi.com/article/10.3390/vaccines10091515/s1, Figure S1: Survey questions for dependent and independent variables.
