# Supplementary material for: Healthcare Provider Recommendations and Observed Changes in HPV Vaccination Acceptance during the COVID-19 Pandemic
Source: Vaccines (Basel). 2022 Sep 12;10(9):1515. doi: 10.3390/vaccines10091515 (PMC9504052; doi:10.3390/vaccines10091515)
Supplement: Supplementary file 1 [file vaccines-10-01515-s001.zip › vaccines-1897993-supplementary.pdf]

Supplementary Material

Figure S1. Survey questions for dependent and independent variables

The next questions are about the impact of Coronavirus (Covid-19) pandemic on HPV vaccination.

During the Covid-19 pandemic, I have observed that

	Increased	Decreased	No change	Not sure
Intention to receive HPV vaccination	◯	◯	◯	◯
HPV vaccination hesitancy	◯	◯	◯	◯
HPV vaccination refusal	◯	◯	◯	◯
HPV vaccination acceptance	◯	◯	◯	◯
HPV vaccination uptake	◯	◯	◯	◯

For the unvaccinated, or incompletely vaccinated for HPV, do you recommend HPV vaccination?

◯ Never
◯ Sometimes
◯ Often/Always
